# Vitamin C and aroma composition of fresh leaves from *Kalanchoe pinnata* and *Kalanchoe daigremontiana*

**DOI:** 10.1038/s41598-019-56359-1

**Published:** 2019-12-24

**Authors:** Renata Zawirska-Wojtasiak, Beata Jankowska, Paulina Piechowska, Sylwia Mildner-Szkudlarz

**Affiliations:** Faculty of Food Science and Nutrition, Poznań University of Life Sciences, Wojska Polskiego 28, 60-637 Poznań, Poland

**Keywords:** Secondary metabolism, Analytical chemistry

## Abstract

Species of kalanchoe are rich in bioactive compounds and are widely used in folk medicine; however, these plants are not well known from the point of view of aroma. Two species, *Kalanchoe pinnata* and *Kalanchoe daigremontiana*, were examined after six months and two years of growth and their vitamin C content, succulence, and aroma composition were determined. The efficiency of juice extraction was highest (72%) for the leaves of *K. daigremontiana* after six months of growth. The concentration of vitamin C was highest in juices from two-year-old plants and much higher in the juice of *K. pinnata* (81 mg/100 g). SPME/GC/MS analysis identified 32 aroma components, considering those with the spectrum similarity over 75%. The main components were furan-2-ethyl, hexanal, 2-hexenal, 2,4-hexadienal, 1-octen-3-ol, nonanal. The quantitative relations of these compounds were somewhat different in the two species. The most dominant component, 2-hexenal, is responsible for the green-like aroma noted by the sensory panel.

## Introduction

According to Gehrig *et al*.^1^ 125 varieties of *Kalanchoe* can be distinguished, divided into 15 taxonomic groups. Those genus belongs to *Crassulaceae*, defined broadly; mostly in Africa, Madagascar, Brazil; several are seen in green houses^2^. The most common in Europe are *K. pinnata* and *K. daigremontiana* (synonyms: *Bryophyllum pinnata* and *Bryophyllum daigremontiana*). The leaves morphology of this two species are different. Those of *K. pinnata* are large, medium fleshy, thick on the short petiole, light green with a red tint. The leaf blade has a serrated crenate margins with inactive buds^3^. The leaves of *K. daigremontiana* are thick, fleshy, lanceolate with pointed shape and purple brown spots on the underside. The most characteristic feature of this species is the method of reproduction consisting in the production of propagates on the crenate margins of the leaves^3^. This species are used in folk medicine in India and other parts of world with a warm climate. *K. daigremontiana* is less used but is easier to cultivate. These plants are rich sources of phenolic compounds, bufadienolides, and vitamins, including ascorbic acid, riboflavin, thiamin, and niacin^4–6^. Pattewar^4^ also indicates that *Kalanchoe* is becoming endangered and needs to be conserved, as well as explored, for its significant green chemistry. This plant might be a promising source of natural antioxidants^7^.

The leaves and juice of *Kalanchoe* possess antimicrobial activity and are used as anti-inflammatory and antiseptic preparations^8–11^, as well as in the treatment of cardiovascular dysfunction^12^, diabetes, and some cancer and chemoprevention^4,12–14^. The medicinal properties of these plants are mostly associated with the very high levels of bioactive compounds such as flavonols, phenolic acid glucosides^15^, flavonoid quercetin, and quercitrin^16^. Ürményi *et al*.^17^ reported of a new triglycoside identified as kaempferol 3-O-*ß*-D-xylopyranosyl-(1→2)-α-L-rhamnopyranoside-7-O-ß-D-glucopyranoside in *K. daigremontiana*. According to authors this plant revealed the therapeutic potential of a plant popularly used against infectious and inflammatory processes. However, the main bioactive compounds in *Kalanchoe* species are bufadienolides and their glucosides which, despite their broad possible medical applications might also be toxic and produce side effects^18^. Therapeutic use of *Kalanchoe* is considerably limited by the lack of clinical evidence. Some observations have been made in experiments with animals, such as chicks^19^; the toxic effects depended on the plant species and dose.

Interesting studies have also been carried out regarding the phytochemical and pharmacological properties of *K. daigremontiana*^20,21^, which are associated with α and β-amyryne, stigmasterol, phenolic compounds, organic acids, alkaloids, and tannins.

Traditional uses of *K. pinnata* inspired El Abdellaoui *et al*.^8^ to seek a method of finding and identifying antimicrobial molecules that could be used instead of common preservatives in cosmetics. The plant’s leaves not only have antibacterial activity, but also act antifungally^22^.

Kruk, Pisarski, and Szymańska^23^ described the isolation of a new form of vitamin E from the green leaves of *K. daigremontiana*; these have been suggested as a possible food additive, but there is little information in the literature on the possible use *Kalanchoe* in nutrition and no information on its flavor and sensory properties. Pattewar^4^ pointed out that the natural ascorbic acid in *Kalanchoe* is vital for the body’s performance—i.e., in the normal formation of intercellular substances throughout the body, including collagen, bone matrix, and dentine. This function of ascorbic acid accounts for its wound-healing properties, and the plant is thus used in herbal medicine for the treatment of common cold and of other diseases, such as prostate cancer.

The high and diverse bioactivity of *Kalanchoe* is important, but from the medicinal point of view, it needs to be excluded from broad uncontrolled use, particularly as a nutrient, because of the possible toxicity^18,19,24^. However, it is still interesting to determine the flavor characteristics (aroma and taste) of *Kalanchoe*, which have barely been studied.

The aim of the work was to obtain juice from the leaves of *Kalanchoe daigremontiana and Kalanchoe pinnata*, to evaluate its aroma constituents, sensory properties, and concentration of vitamin C.

## Materials and Methods

### Samples

The study was conducted with two species of *Kalanchoe*, namely *K. pinnata* and *K. daigremontiana*. The *K. pinnata* material was received from Poznań Botanical Gardens (catalogue number 8586/11) and propagated by us, while the *Kalanchoe daigremontiana* was obtained from an ecological nursery (Rafał Figas, Mochnaczka Wyżna, Mazowieckie, Poland). The cultures were grown in culture room with temperature 21 ± 2 °C and natural light without fertilization. When growing the plants received water in moderate amounts, not frequent than once a week. The fully expanded leaves were cropped after six months and after twenty-four months of cultivation, before flowering.

The juices were obtained by crushing fresh washed leave without petioles and then room-temperature pressing using laboratory cold-pressing machine. After passing through filtration material were stored in closed, fully filled bottles in the dark at 4 °C. The efficiency of this process was calculated from the weight of leaves and juice. The juice from leaves of *K. daigremontiana* was more green in color.

### Estimation of dry matter in *Kalanchoe* leaves

Dry matter was estimated in line with Polish norm PN-EN 12145:2001^25^.

### Estimation of vitamin C contents

Vitamin C was estimated in juices from the leaves of *Kalanchoe* with Tillmans’ reagent^26^.

### SPME/GC/MS analysis of volatiles

Levels of volatile were determined in the leaves, juice, and pulp residue of *Kalanchoe pinnata* and *Kalanchoe daigremontiana*. The analysis was carried out using the SPME/GC/MS method with a 75 µm Carboxem-PDMS fiber (Supelco Bellefonte, PA, USA). The parameters of microextraction were optimized to 30 min and 50 °C; 2 g leaves, 2 g juice, or 5 g residue were weighed into 20 ml vials. After 30 min exposure, the fiber was removed from the vial to the injection port of the gas chromatograph for 5 min of desorption. GC separation was performed on an Agilent 7890 A gas chromatograph with an Agilent VLMSD detector and Supelcowax-10 column (30 m × 0.3 mm × 0.25 µm). Compounds were separated using the following temperature program: from 45 °C (hold 2 min) to 50 °C at a rate of 1 °C/min, then to 170 °C at a rate of 8 °C/min, and finally to 230 °C at a rate of 18 °C/min (held 8 min). The carrier gas was helium, the detector temperature was 270 °C, and the injector temperature was 220 °C.

The separated compounds were identified by comparing their retention indices (RI) and mass spectra with standards or, in some cases, tentatively through a search of the NIST MS Search 2.0 mass spectra library, considering those with spectrum similarity over 75%. Quantitative estimation was done on the basis of peak areas and expressed as percentage contribution.

### Sensory analysis

The triangle method was used to compare samples of water with the addition of *K. daigremontiana* juice (0.2%, 0.5%, 1.0%, 2.0%, 5.0%, and 10.0%) to pure water. Samples codes were generated according to Baryłko-Pikielna & Matuszewska^27^. A twelve-member panel considered 24 samples (the six possible triangles were presented in four repetitions), and the data was read out from statistical tables for the triangular method^27^.

A preference test was carried out with the same twelve-member panel to determine the most accepted concentration of juice (5%, 10%, 30%, or 50%). They rated the taste and smell of juice presented in 30 ml samples in closed black vessels at room temperature. When tasting, the panelists were asked to not swallow the juice. They ranked the samples in order from least to most accepted.

The sensory aroma profile was carried out using a previously developed list of attributes (Table 1), which were selected in a special session with the *Kalanchoe daigremontiana* juice samples. The attribute descriptions were taken from Stampanoni Koeferli^28^. The profile was developed for juice (10%, 30%, and 100%) from the leaves of *K. daigremontiana* that had been cultivated for 24 months: additionally, 100% juice from six months of cultivation was also introduced.

**Table 1:** Sensory attributes selected for flavor profile analysis of *Kalanchoe daigremontiana* juice (Stampanoni Koeferli, 1998).

No	Attribute (smell)	Description
1.	GREEN	Characteristic for fresh cut grass or green leaves
2.	CUCUMBER	Characteristic for fresh cut cucumber
3.	KIWI, MELON	Characteristic for fruits of kiwi or melon
4.	HERBAL	Characteristic for mixture of herbs: basil, lovage, thym in form of essential oils or fresh leaves
5.	EARTHY	Reminiscent raw (no washed) potato or raw red beetroot
	**Attribute (taste)**	**Description**
6.	BITTER	Basic taste, caused by (0,5-1%) water solution of caffeine I or quinine
7.	ACIDIC	Basic taste caused by (1%) water solution of citric, tartaric, lactic, characteristic for fermented products
8.	ASTRINGENT	Giving characteristic sensation for water solution of tannin, strong tea, red wine (Bordeaux), green banana

## Results and Discussion

According to de Araújo *et al*.^29^. *Kalanchoe* leaves and their juice can be used as raw materials in medical treatment. At the start of the experiments, the juice was prepared from leaves of *K. daigremontiana* and *K. pinnata*. The efficiency of the juice extraction process depended on the species and age of the plant. The highest value (72%) was obtained for juice prepared from *K. daigremontiana* after six months of growth, while 58% was the efficiency in the case of juice from the two-year-old plant; scores of 43% and 32% were obtained for *K. pinnata*. *K. daigremontiana* provided more juice, but the leaves of this species contained twice as much moisture (Table 2).

**Table 2:** Concentration of Vitamin C and juice efficiency for *Kalanchoe* leaves.

Variety	Age months	Dry mass (%)	Juice extraction efficiency (%)	Vit.C* mg/100 g of juice	Vit.C* mg/100 g of juice after 1 week of storage
*Kalanchoe* *daigremontiana*	6	3.47	72.11	9	6
*Kalanchoe* *daigremontiana*	24	4.29	58.27	18	12
*Kalanchoe* *pinnata*	6	9.04	43.21	72	57
*Kalanchoe* *pinnata*	24	9.73	32.53	81	66

*Mean value of 3 repetitions, coefficient of variation 2.9%.

We estimated the levels of vitamin C in the juices. Pattewar^4^ pointed out the importance of natural ascorbic acid in *Kalanchoe* species. Other literature sources have also suggested that they contain significant concentrations of vitamin C, but did not show any quantitative data. A very high concentration of vitamin C was found in the juice of *K. pinnata—*more than four times higher than in the juice of *K. daigremontiana*; 18 mg/100 g of juice from the two-year-old plant and 81 mg/100 g respectively (Table 2). Independently of species, the concentration of vitamin C was much higher in the juice from the two-year-old plants than in that from the six-month plants.

The concentration of vitamin C in *Kalanchoe* juices corresponds to the level in blackcurrant products: Mattila *et al*.^30^ examined the concentrations of vitamin C in commercial blackcurrant juices and found that it ranged over a relatively wide spectrum, depending on the European country of origin (e.g., 70 mg/2.5 dl in juice from the UK and 15 mg/2.5 dl in Finnish juice). In this study, the value for juice from *K. daigremontiana* was close that of Finnish blackcurrant products, while the value for the juice of *K. pinnata* was similar to that of UK blackcurrant products.

Vitamin C is not stable during storage or processing^31^, so after the experiment was conducted, the juices were stored for one week at 4 °C: 23% and 33% of vitamin C was lost from the six-month and two-year juices of *K. daigremontiana*, respectively, and 20.8% and 18.5% respectively in the case of *K. pinnata* (Table 2). This means that the juice would better be used fresh.

Sensory analysis was performed on the juice from *K. daigremontiana*, which is more common in Poland (as sufficient material could be obtained for this species). The main purpose of the sensory measurements was to determine the acceptable concentration of *Kalanchoe* juice. Triangle tests were used to determine the difference between the aroma of the control samples (pure water) and water with addition of 0.2%, 0.5%, 1.0%, 2.0%, 5.0%, and 10% of juice. On the basis of 24 repetitions (triangles) and the statistical tables for this method^27^, even the lowest concentration of juice was differentiated at a statistical significance of α = 0.05. The samples with the other added amounts were detected for significance values as low as α = 0.001.

Using the preference method, the twelve panelists tested the taste and aroma of samples consisting of water with the addition of 5%, 10%, 30%, and 50% *K. daigremontiana* juice. The most preferred in terms of smell were the 50% addition and the 30% addition; in terms of taste alone, the 10% addition was preferred.

**Figure 1:**
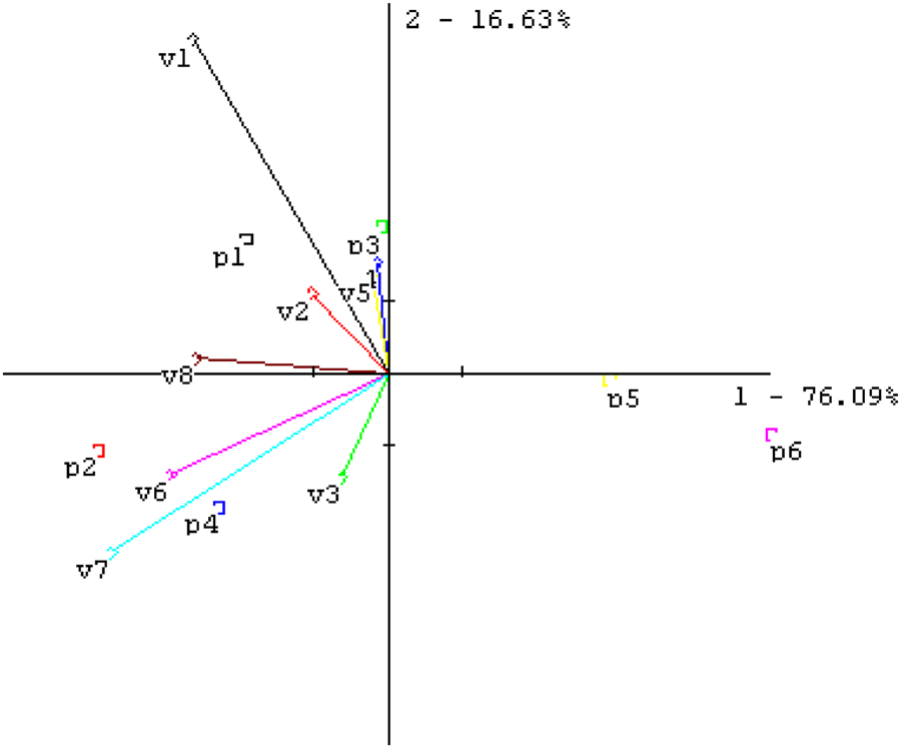
PCA plot of sensory profile data for samples of *Kalanchoe daigremontiana* juices. Sample codes: p1 – juice I 100% 6 months, p2 – juice I 100% 2 years, p3 - juice II 100% 6 months, p4 – juice II 100% 2 years, and p5 – juice II 30% 2 years, p6 – juice II 10% 2 years. Descriptors: v1 – green, v2 – cucumber, v3 – kiwi, melon like, v4 – herbal, v5 –earthy, v6 –bitter taste, v7- sour taste, v8 – astringent.

Sensory profile analysis of smell and taste was performed for six samples of *K. daigremontiana* juice*;* two 100% juices were prepared from a plant that had grown for six months, and two from the 24-month plant; these were added at a rate of 30% and 10% (from the 24-month plant) to water. A PCA graph of the data is presented in Fig. 1. The most discriminating attributes were green smell (v1) and sour taste (v7). The 100% juice samples (p1, p2, p3, p4) were very aromatic, but also sour (v7) and bitter in taste (v6)—particularly those from the two-year-old plant (p2 and p4); the samples of juice from the six-month plant (p1 and p3) were more green-like (v1) in character. Two samples (p5 and p6) that consisted of only 30% and 10% juice in water were not aromatic, but were also not bitter or sour. All measurements point to no strong aroma that disappeared in dilution.

**Figure 2:**
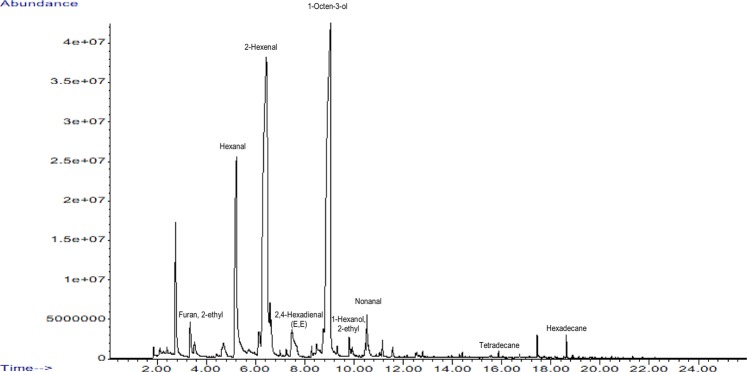
Chromatogram of GC/MS separation of volatiles from juice of *Kalanchoe pinnata*.

**Figure 3:**
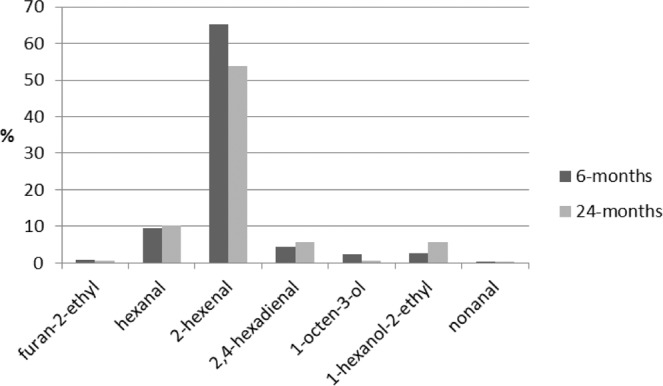
Percentage composition of the 6 main volatiles in the sample of *Kalanchoe daigremontiana*.

**Figure 4:**
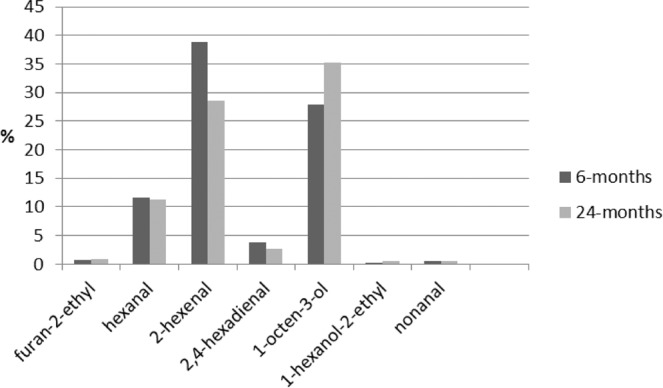
Percentage composition of the 6 main volatiles in the sample of *Kalanchoe pinnata*.

The main goal of the study was to determine *Kalanchoe* volatiles using gas chromatography. From all the samples of *K*. *daigremontiana* and *K. pinnata* leaves and juice, the SPME/GC/MS analysis identified 31 components, considering those with a spectrum similarity over 75%. (Table 3). Figure 2 presents the gas chromatogram of one selected sample for the juice of *K. pinnata*. More of the compounds occurred in samples of *K. pinnata*, and almost all of them in the two-year-old plant. About ten of these compounds occurred only in samples of *K. pinnata*. Among those identified only in *K. pinnata* were 1,3-octadiene, octanal, 2-octenal (E), 2-octen-1-ol (E), 2-octen-1-ol, and dodecane. On the other hand, furan-2-pentyl and 1,4-hexadiene-3-ethyl, were identified only in *K. daigremontiana*. Nine components occurred in almost all samples of both varieties: furan-2-ethyl, hexanal, 2-hexenal, 2,4-hexadienal, 1-octen-3-ol, 1-hexanol-2-ethyl, nonanal, tetradecane, and hexadecane. The last two are paraffins, and not very significant for the aroma. Dominant among the rest was 2-hexenal, at about 70% of total components in the juice of the six-month-old *K. daigremontiana* plant and over 38% in the juice of six-month-old *K. pinnata* (Figs. 3 and 4), but a little less in the two-year-old plant. This composition explains the dominant green smell found in sensory analysis of the *K. daigremontiana* juices, which were associated with hexanal and hexenal. One characteristic of *K. pinnata* was 1-octen-3-ol which occurred in both varieties, but in significant amounts only in *K. pinnata*, where it amounted to 35% in the juice from the two-year-old plant.

**Table 3:** Volatiles identified in samples of K*alanchoe daigremontiana* and *Kalanchoe pinnata*.

RT (min)	Name of compound	*K.d*. 6 months		*K.d*. 2 years		*K.p*. 6 months		*K.p*. 2 years	
		leaves	juice	leaves	juice	leaves	juice	leaves	juice
3.33	1-Penten-3-one				X			X	
**3.51**	**Furan, 2-ethyl**	**X**	**X**	**X**	**X**		**X**	**X**	**X**
**5.21**	**Hexanal**	**X**	**X**	**X**	**X**	**X**		**X**	**X**
5.71	1,3-Octadiene					X	X		
**6.72**	**2-Hexenal**	**X**	**X**	**X**	**X**	**X**	**X**	**X**	**X**
6.98	2-Heptanone								X
7.27	Heptanal	X		X				X	X
**7.49**	**2,4-Hexadienal (E,E)**	**X**	**X**	**X**	**X**	**X**	**X**	**X**	**X**
8.29	2-Heptanone, 6-methyl							X	X
8.48	2 (5 H) – Furanone,5-ethyl			X		X	X		X
8.50	Oxalic acid		X					X	
**8.88**	**1-Octen-3-ol**	**X**	**X**	**X**		**X**	**X**	**X**	**X**
9.02	Furan, 2-pentyl	X		X					
9.19	1,4- Hexadiene,3-ethyl			X	X				
9.31	Octanal						X	X	X
**9.80**	**1-Hexanol, 2-ethyl**	**X**	**X**	**X**	**X**			**X**	**X**
10.34	2-Octenal (E)						X	X	
10.53	2-Octen-1-ol (E)						X		X
**11.15**	**Nonanal**	**X**	**X**	**X**	**X**	**X**	**X**	**X**	
12.15	2-Nonenal (E)		X					X	
12.55	Cyclohexanol,5-methyl-2-(1-methylethyl)		X		X	X	X		
12.89	Decanal	X		X		X		X	
12.95	Dodecane					X		X	
13.24	1-Cyclohexene-1-carboxaldehyde,2,6,6-trimethyl	X		X				X	
14.37	Tridecane					X		X	
**15.86**	**Tetradecane**	**X**	**X**		**X**	**X**		**X**	**X**
17.29	Pentadecane					X		X	
17.44	Butylated Hydroxytoluene						X		X
17.49	Phenol, 2,4-bis (1,1-dimethylethyl)						X		X
18.52	Pentanoic acid				X	X	X		
**18.63**	**Hexadecane**	**X**	**X**		**X**	**X**	**X**	**X**	**X**

RT – Retention Time X – identified by GC/MS with 75% of spectrum similarity.

*K.d. Kalanchoe daigremontiana, K.p. Kalanchoe pinnata*.

6 months – plant growing 6 months 2 years – plant growing 2 years.

In last published paper by Obregón-Díaz^32^, the author identified nineteen compounds in essential oil from *K. pinnata*, obtained by hydrodistillation. Among them one of the main volatile compound was 1-octen-3-ol representing 18.1% of the oil. 1-octen-3-ol did not give a mushroom-like effect in the aroma profile of the samples from *K. daigremontiana*, which contained very small amounts of it. According to Almeida, Muzitano & Costa^33^ 1-octen-3-ol occurs in the leaves of *K. pinnata* as an aglycone of a minor vinylic aliphatic alcohol diglycoside with the proposed structure: 1-octen-3-O-α-L-arabinopyranosyl—(1→6)-ß-glucopyranoside. The presence in the aroma of *K. pinnata* of a few other eight-atom compounds (mentioned above) not found in *K. daigremontiana* is interesting. All these observation suggest that, despite some similarities—such as the dominance of 2-hexanal in both species—there are also significant differences in aroma composition, such as the mushroom-like component in *K. pinnata*., which is probably the key compound in its perceived aroma.

**Figure 5:**
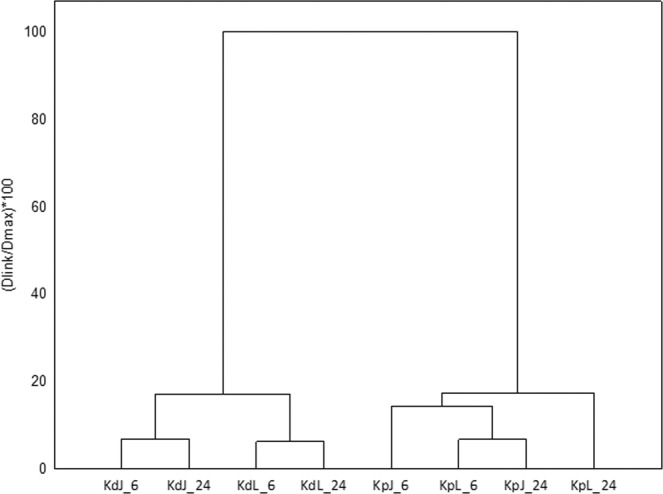
Dendrograms for the hierarchical cluster analysis (HCA) results using Ward’s cluster algorithm for the dataset of *Kalanchoe* species showing their aromatic composition. Sample codes: leaves of *K. daigremontiana* after 6 or 24 months (KdL_6 and KdL_24), juice of *K. daigremontiana* after 6 or 24 months (KdJ_6 and KdJ_24), leaves of *K. pinnata* after 6 or 24 months (KpL_6 and KpL_24), juice of *K. pinnata* after 6 or 24 months (KpJ_6 and KpJ_24).

Hierarchical cluster analysis of the data on volatile compounds shows that two groups are observed (Fig. 5). Two *Kalanchoe* species of *K. pinnata* and *K. daigremontiana* were characterized by several volatile components constituted two distant clusters. As seen in the dendrogram in Fig. 5 the first cluster consists of the samples of *K. daigremontiana* with two sub-groups formed by juice and leaves samples. The second cluster contains samples of *K. pinnata*. It confirm the differences of aroma profile between two studied species, however the variation inside the clusters is more complicated *in K. pinnata*.

## Conclusion

The leaves of *Kalanchoe daigremontiana and Kalanchoe pinnata* are juicy and rich in vitamin C, but do not possess an intense aroma. The occurrence and relations of aroma compounds depended on the species, but less on the age of plant. The sensory measurements for *K. daigremontiana* showed a pleasant accepted aroma, in contrast to a sour and bitter taste. However the taste would seem to be of no importance, because **Kalanchoe** cannot be used as a nutrient due to the risk of development of cardiotoxic effects, or perhaps not even as an oral medicine, but only for the treatment of skin diseases and also in cosmetics. For this latter application, the pleasant green-like aroma of *K. daigremontiana* may be useful; however, it is not very strong, and is perceived only in higher concentrations. The broader use of these valuable plants is not possible at the moment, but they are worth further investigations aimed at elucidating the effects of the bufadienolides on health, and determining their dose limits.
